# Efficacy of eHealth interventions to reduce depression symptoms in individuals with obesity: a systematic review of randomized controlled trials

**DOI:** 10.3389/fpsyt.2024.1296433

**Published:** 2024-03-07

**Authors:** Dilara Kocol, Alexander Bäuerle, Theresa Schadendorf, Sheila Geiger, Julia Barbara Krakowczyk, Eva-Maria Skoda, Martin Teufel

**Affiliations:** ^1^ Clinic for Psychosomatic Medicine and Psychotherapy, LVR-University Hospital Essen, University of Duisburg-Essen, Essen, Germany; ^2^ Center for Translational Neuro- and Behavioral Sciences (C-TNBS), University of Duisburg-Essen, Essen, Germany

**Keywords:** e-mental health, depression, eHealth intervention, obesity, mental health

## Abstract

**Introduction:**

Obesity and depression are inter-related health concerns, demanding a high level of treatment and costs in the health care system. The development of eHealth interventions that simultaneously address obesity and mental health can be supportive in this regard. However, evidence of the efficacy of eHealth interventions in the treatment of depression symptoms in individuals with obesity is lacking. The aim of this systematic literature review is to evaluate the efficacy of existing eHealth interventions for individuals with obesity that target depression symptoms.

**Methods:**

We systematically searched electronic databases (Cochrane Library, PubMed, Scopus) to identify studies published in English between January 2016 and January 2023, that focused on eHealth interventions, targeting depression symptoms in individuals with obesity people. Exclusion criteria were study objectives that (1) focused specifically on one or more metabolic comorbidities of individuals with obesity, e.g., hypertension, hyperlipidemia, diabetes; (2) focused specifically on eating disorders comorbidities e.g., binge eating disorder, and (3) focused specifically on patients before or after bariatric surgery.

**Results:**

The database search identified 214 records. Six articles were included in this review. Sample sizes ranged from 70 to 1267 participants of ages 18-60 years. All included studies were randomized controlled trials. Two of the six included studies were web-based interventions guided either by medical doctors or psychologists. All interventions included video, printed materials, and interactive parts of which two studies integrated elements of Cognitive Behavioural Therapy and Social Cognitive Therapy. The findings showed that eHealth treatment services, supported and guided throughout the intervention had high acceptance and efficacy in the reduction of depression symptoms among individuals with obesity.

**Conclusion:**

EHealth interventions that address and target both mental and physical health with interactive strategies calls for better efficacy in the reduction of depression symptoms. Future eHealth interventions that target depression symptoms in individuals with obesity should integrate digital strategies that address both mental and physical health through interactive modules.

## Introduction

One of the most emerging health issues and prevalent chronic diseases worldwide can be ascribed to overweight and obesity (1). Overweight and obesity are defined as abnormal or excessive fat accumulation that can cause several health issues (1). The Body mass index (BMI) is an index of weight-for-height, used to classify overweight and obesity in adults (1). Hereby, a BMI greater than or equal to 25 defines overweight and a BMI greater than or equal to 30 obesity (1). The so-called obesity pandemic has become a significant global health challenge (1). According to data of the World Health Organisation in 2016, around 1.9 billion of people 18 years and older were overweight, of which over 650 million were obese (1). Hereby, the global prevalence of individuals with obesity has almost tripled between the years 1975 and 2016. Current data shows that obesity and overweight turned out to be increasingly prevalent in low- and middle- income countries, in particular in urban areas (1). Based on data from the European Health Interview Survey EHIS conducted in 2019, it became apparent that around 36% of adults in EU Member States were overweight and 17% were obese (2). The outcomes of the Global Burden of Disease study (GBD) conducted in 2017 showed that a BMI above 25 caused 2.4 million deaths in females and 2.3 million deaths in males worldwide. Next to the global deaths, the GBD study analyzed the disability-adjusted life years (DALYs) that can be ascribed to obesity. Hereby, it became apparent that obesity led to 70.7 million DALYs in females and 77.0 million in males. Looking at the number of global deaths and DALYs between 1990 and 2017 in the study showed that the global number of obesity-related DALYs more than doubled in both, men and women (3).

Obesity is associated with an enhanced risk of type 2 diabetes, cancer, stroke, arthritis (4), hypertension, dyslipidemia, cardiovascular risks and musculoskeletal disorders (5). Furthermore, existing data showed that obesity is linked to several mental health problems, especially depression (6–8). It was highlighted that depression and obesity commonly co-exist (9), which was emphasized by a conducted meta-analysis that detected a complex reciprocal pathway between these two diseases (10). A study conducted by Zhang (2021) focused on the bidirectional relationship between body weight and depression and possible explanatory factors and models for this relationship. The outcomes of a psychosomatic-psychotherapeutic evaluation conducted with individuals with obesity showed that one or more mental disorders, especially depression and adjustment disorders, were detectable in more than 50% of the patients (11). Next to these, individuals with obesity experience problems in emotion and behaviour regulation (12), difficulties with peers (13), lower self-esteem (14), and body dissatisfaction (15).

This increase of obesity and the related comorbidities associated with it contribute to high health costs. The results of systematic reviews show that between 1.9% and 4.7% of total annual healthcare costs, and 2.8% of total annual hospital care costs in European countries are attributable to obesity (16, 17).

Due to this high incidence and comorbidity of the double stigma obesity and mental health issues experienced by individuals with obesity (18–20), several mental health research experts proposed to label obesity as a mental disorder in the DSM-V (20, 21). This has led to the establishment of overweight and obesity treatment programs that aim to have, besides the impact on the primary goal of weight management, an influence on the mental health as well (18). Interventions that integrate elements of cognitive behavioural therapy (CBT) have been shown to lead to positive outcomes in improving mental health (22). In general, most mental health interventions for individuals with obesity aim to increase knowledge about potential difficulties associated with dieting by simultaneously providing psychoeducation about weight biology, increased self-esteem and mental health, improved health and eating behaviours, and physical activity, rather than focusing predominantly on weight loss (23, 24).

The high global prevalence of obesity that is highly connected to further physical comorbidities and mental health difficulties (25) requires a high and long-term treatment effort that has financial consequences and leads to an increased burden on the health care system (26, 27). The outcomes of face-to-face treatments in these contexts showed that there was deficient proof of the “scalability, generalisability and long-term sustainability” of face-to-face treatments (28) which opened at that point doors to the establishment of eHealth interventions, including approaches to improve the mental health of individuals with obesity.

EHealth interventions in general can be defined as the usage of information and communication technologies (ICT), aiming an improvement and facilitation in the health care system and health conditions (29). Furthermore, it fosters an option to “enhance the quality, efficiency, and reach of primary and secondary healthcare” (30) in a timely and cost-effectively more flexible way for practitioners and patients (31). In particular, the outcomes of a study conducted by Bauerle et al. (32) emphasized the increased mental health burden during the COVID-19 pandemic and the need for appropriate interventions to support burdened people. Due to the new health system realities created by the COVID-19 pandemic, low-threshold tele-medical approaches provide opportunities in anonymous and effective support and treatment (33).

Several systematic reviews provide evidence for the effectiveness of eHealth interventions in the treatment of individuals with obesity (34–36). The outcomes of a review emphasized the positive impact of Internet interventions on improving disease knowledge, health outcomes, health care utilization and quality of life (37). Based on the gained insights and evidence about the co-existence of obesity and depression, electronic lifestyle programs were developed that target physical and mental health simultaneously (38). These programs also provide success in weight and depression symptom reduction. Due to the fact, that such digital lifestyle programs focusing on individuals in a holistic way have been proven to be effective (39, 40), more eHealth interventions in obesity treatments have been developed (41, 42). The results of a self-guided, eHealth program targeting weight loss and depression in men showed a significant decrease in weight and depression (42).

Despite the emerging empirical evidence highlighted above regarding the inclusion of mental health approaches that specifically target high-prevalence depression in obesity and its electronic treatment programs, there is no clear synthesis of the literature. This lack of understanding leads to limited efforts to address and protect depression symptoms in obesity interventions (43). Hence, it is of relevance to have a clear synthesis of evidence relating to the efficacy of eHealth interventions targeting depression symptoms in individuals with obesity. Therefore, the research question in this systematic literature review is:

How does the efficacy of eHealth interventions, targeting depression symptoms in individuals with obesity compare to a control condition in randomized controlled intervention trials?

## Materials and methods

This systematic literature review was performed following the PRISMA guidelines (44).

### Search strategy

A comprehensive literature search of the Cochrane Library, PubMed, and Scopus databases was conducted between November 2022 and January 2023. The search results were included into the analyses until January 31, 2023. Additionally, the so-called snowball-method was applied. This involved searching the reference lists of relevant articles for potential articles of potential interest. Further, previously conducted systematic reviews focusing on eHealth interventions in obesity treatment were also screened. The literature search was limited to studies published between January 2016 and January 2023. Two independent researchers conducted the literature search. The following combinations of search terms were used: obes* AND randomized controlled trial AND depress* AND e-health OR mobile OR e-mental health OR web OR tele* AND adult. All included studies were RCTs because RCTs best isolate the effects of a particular intervention and provide evidence for or against a particular intervention. RCT’s represent the gold standard in clinical research. The relevant syntax for each searched database, including the number of results found for each database, are provided in a **Supplementary Table 1**.

### Inclusion/exclusion criteria

The search was designed to identify studies of eHealth interventions, targeting depression symptoms in individuals with obesity. All published (RCTs) of eHealth interventions to reduce depression symptoms, compared with a control condition targeting individuals with obesity were eligible for inclusion in this systematic review. Inclusion criteria were (1) individuals with obesity (BMI = 30 kg/m²) targeted, independent of a specific topographic limitation, ethnicity, or occupational group; (2) an eHealth intervention targeting depression symptoms; (3) a mental health measurement of depression symptoms reported at baseline and post-intervention; (4) a control group; (5) publication in English between January 2016 and January 2023. Exclusion criteria were (1) study objectives that focused specifically on one or more metabolic comorbidities of individuals with obesity, e.g., hypertension, hyperlipidemia, diabetes; (2) focused on eating disorders comorbidities e.g., binge eating disorder, and (3) focused specifically on patients before or after bariatric surgery.

### Data extraction and data synthesis

**Figure 1:**
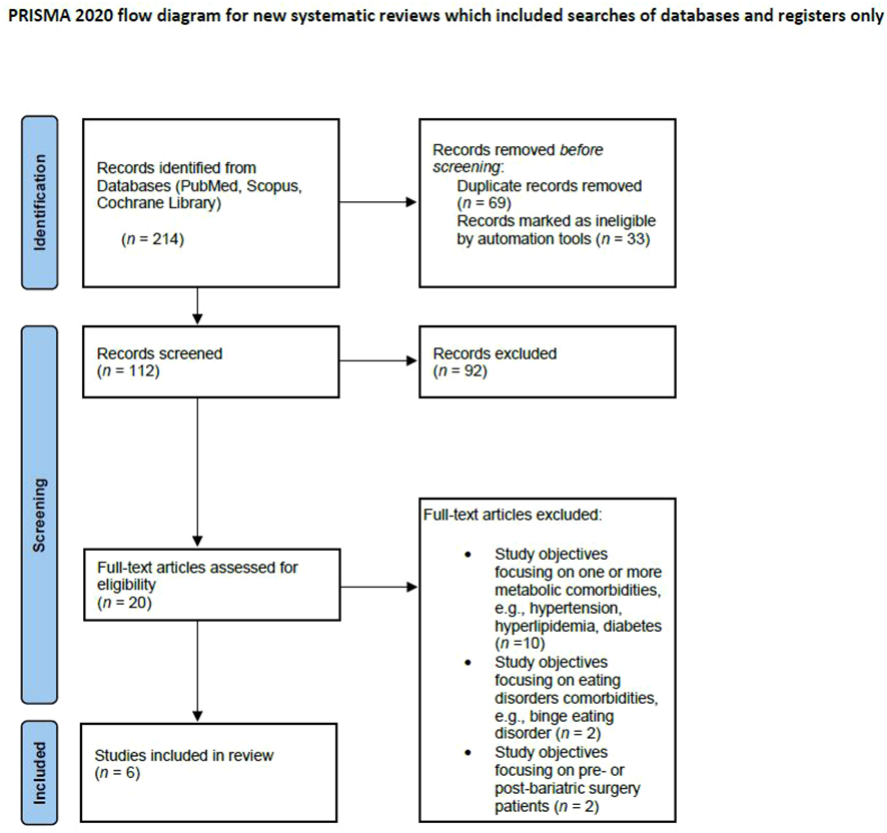
Flow diagram of studies that were identified using the search terms and strategy, articles screened for eligibility, included/excluded with reasons following PRISMA guidelines.

Data extraction was performed independently by D.K and T.S. Thirteen articles were selected for full-text review to assess eligibility for inclusion. A standardized data extraction form was created to capture sample characteristics, intervention characteristics, eHealth approach, measures used in general and measures specifically targeting depression, and findings (**Table 1**). The authors resolved discrepancies by consensus. The authors first screened titles and abstracts of each article. A list of potential articles was further shortened by reviewing abstracts and performing detailed evaluations by considering the above-mentioned eligibility criteria. See **Figure 1** for the progressive flow of the study exclusion process.

**Table 1:** Summary of included eHealth interventions to reduce depression in obese people.

Study + *keywords*	Sample characteristics	Intervention characteristics + eHealth approach	Measures	Findings
**Chang et al.** (45) *Mothers in Motion Intervention effect on psychosocial health in young, low-income women with overweight or obesity * *keywords:* low-income women, stress, depression, obesity	Mean BMI (kg/m^2^): 32.0 Mean age (years): 28.5 Gender (% female): 100.0 *N* = 569	→ 16-week intervention, integrating approaches applied in Cognitive Behavioral Therapy (CBT): Intervention group: ◦10 video lessons (DVD format) ◦Peer support group teleconferences (30 min per session) ◦Both weekly for the first four weeks, then every other week for weeks 5–16 Control group: ◦Printed learning materials	Depression: •Center for Epidemiologic Studies-Depression Scale (CES-D) Other: ◦Perceived Stress Scale (PSS) ◦Positive and Negative Affect Scale (PANAS) ◦Factors Affecting Diet, Exercise, and Stress Management (FADESM) Scale	Findings – Depression: •Group differences in favor of the intervention group for depression Findings – Other: ◦Group differences in favor of the intervention group: ◦Self-efficacy to cope with stress ◦Emotional coping response Stress ◦Positive affect No group differences: ◦Social support for stress management ◦Negative effect
**Jones et al.** (46) *The impact of participant mental health on attendance and engagement in a trial of behavioural weight management programmes * *keywords:* Obesity, Prevention, Weight loss, Mental health, Engagement	Mean BMI (kg/m^2^): 34.5 Mean age (years): 53.2 Gender (% female): 68.0 *N* = 1267	→ Brief intervention, integrating approaches applied in Cognitive Behavioral Therapy (CBT): ◦Printed learning materials → 12-week commercial weight management program → 52-week commercial weight management program ◦Access to Weight Watchers (WW) digital tools and (online) resources (e.g., recipes, videos, community area) ◦Mobile phone application	Depression: •Hospital Anxiety and Depression Scale (HADS) Other: ◦European Quality of Life 5 Dimensions (EQ5D) ◦Satisfaction with Life Scale (SWLS)	Findings – Depression: •Higher baseline depression ⇢ reduction in WW program attendance •Lower levels of depression ⇢ higher attendance at study visits Findings – Other: ◦Higher baseline anxiety ⇢ reduction of the likelihood of self-reported high engagement with the program (WW e-tools, mobile app) ◦Higher baseline global quality of life ⇢ lower odds of reporting high engagement with the mobile app ◦Higher scores for quality of life, satisfaction with life ⇢ higher attendance at study visits ◦Lower levels of anxiety ⇢ higher attendance at study visits
**Kim et al.** (47) *Multidimensional Cognitive Behavioral Therapy for Obesity Applied by Psychologists Using a Digital Platform* *keywords:* Obesity, Digital health care, Cognitive behavioral therapy, Mobile phone	Mean BMI (kg/m^2^): 28.0 Mean age (years): 21.8 Gender (% female): 100.0 *N* = 70	→ 8-week intervention Intervention group: ◦Noom app, integrating approaches applied in Cognitive Behavioral Therapy (CBT): ◦Log food intake, exercise activities, weight ◦In-app daily individual feedback, assignments from a psychologist ◦In-app weekly group activities ◦Deliver in-app daily report, weekly report, midweek report to the participants to enhance goal setting and motivation ◦Web-based dashboard available to therapist to monitor data InBody H20B analyzer ◦Monitors and collects body composition data of the participants Telephone Interview (in case of inactivity) Control group: ◦Practicing self-care without therapist intervention	Depression: •Korean Beck Depression Inventory-II (K-BDI-II) Other: ◦Situational Motivational Scale (SIMS) ◦Body Shape Questionnare-8C (BSQ-8C) ◦Trait Anxiety Inventory (TAI) ◦Rosenberg Self-Esteem Scale (RSES) ◦Anthropometric measures (e.g., BMI, weight) ◦Blood measures ◦Dutch Eating Behavior Questionnaire (DEBQ) ◦Automatic Thoughts Questionnaire (ATQ-30) ◦Yale Food Addiction Scale (YFAS)	Findings – Depression: •Baseline depression predicts long-term clinical outcomes. Findings – Other: Group differences in favor of the intervention group: ◦Weight loss ◦Fat mass reduction ◦Mean leptin and insulin resistance ◦Emotional eating behavior ◦Mean Snack calorie intake →Baseline anxiety and self-esteem levels predict long-term clinical outcomes. →Baseline motivation predicts short-term and long-term outcomes.
Marcus et al. (48) *Pasos Hacia La Salud: a randomized controlled trial of an internet-delivered physical activity intervention for Latinas* *keywords:* Physical activity, Latinas, Internet, Technology, Behavioral intervention, Public health	Mean BMI (kg/m^2^): 28.8 Mean age (years): 39.2 Gender (% female): 100.0 *N* = 205	→ 24-week intervention, integrating approaches applied in Cognitive Behavioral Therapy Intervention group: ◦Self-monitoring of activity and steps ◦Goal setting with graphics ◦Message board to allow social support between participants ◦Question tool (contact to researchers) ◦Online resources (e.g., exercise videos, educational templates) ◦Individualized online manuals (e.g., goal setting, problem solving) ◦Links to online and community resources ◦Monthly questionnaires ◦Mail prompts weekly during month 1, bi-weekly during months 2 and 3, and monthly during months 4–6 Control group: ◦Content focused on diet, cardiovascular diseases, heart health ◦Same number of email contacts as the Intervention Group ◦Monthly questionnaires	Depression: •CES-D Other: ◦PSS Social Support Scales ◦Physical Activity Enjoyment Scale (PACES) ◦Neighborhood Environment Walkability Scale (NEWSA) ◦Short-Test of Functional Health Literacy in Adults (S-TOFHLA) ◦Seven-day physical activity recall (7-Day PAR) ◦Accelerometer-measured physical activity (ActiGraph 3X+) ◦Stage of change ◦Scale ◦Self-efficacy for physical activity (SEPA) scale ◦Processes of Change Scale (POC)	Findings – Depression: •No group differences regarding depression Findings – Other: Group differences in favor of the intervention group: ◦Min/week of Moderate-to-vigorous physical activity (MVPA) ◦Meet national physical activity guidelines ◦Self-efficacy ◦Cognitive processes ◦Behavioral processes ◦Trend for enjoyment No group differences: ◦Changes in social support Perceived stress
Welzel et al. (49) *Using a brief web-based 5A intervention to improve weight management in primary care * *keywords:* Obesity, 5As counseling, Primary care, Provider-patient-interaction, CRCT	Mean BMI (kg/m^2^): 39.0 Mean age (years): 43.3 Gender (% female): 62.2 *N* = 135 Participants were recruited from 50 general practices (GP), with general practitioners divided into intervention and control practices. The recruited GPs had a mean age of 48.6 years and were 61.2 % female. GPs in the intervention practices had higher obesity stigma scores.	→ 40-minute intervention, , integrating approaches applied in Acceptance and Commitment Therapy (ACT): Intervention group: ◦5A framework = Online tool for primary care physicians to improve weight management of primary care patients. ◦Five knowledge sections (discussing weight, assessing the patient adequately, information on health benefits and available treatment options, agreeing on treatment goals, assisting with weight-management) ◦Knowledge quiz Control group: ◦Treatment as usual (determined by GP)	Depression: •German Patient Health Questionnaire – 9 (PHQ-9) Other: ◦PHQ-D subscales (panic syndrome, other anxiety syndrome) ◦Big Five Inventory (BFI-10) ◦German Patient Assessment of Chronic Illness Care (PACIC 5A) ◦BMI ◦German EQ-5D-5L ◦German Weight Bias Internalization Scale (WBIS) ◦Readiness Ruler (adapted version) ◦Stages of change algorithm (adapted version) ◦Counseling experiences (4 generated items) For the GPs: ◦German adaption of the short Fat Phobia Scale (FPS) ◦Generated items investigating GPs believes and adjustments ◦Items evaluating the online intervention and its usability	Findings – Depression: •No group differences regarding depression Findings – Other: ◦Group differences in favor of the intervention group: ◦Self-stigma (WBIS) No group differences: ◦Patient assessment of chronic illness care ◦Weight ◦Quality of life Group differences not in favor of the intervention group: ◦Readiness for weight management
**Young et al.** (42) *Impact of a self-guided, eHealth program targeting weight loss and depression in men * *keywords:* low mood, obesity, males, intervention, online	Mean BMI (kg/m^2^): 32.6 Mean age (years): 48.4 Gender (% female): 0.0 * N* = 125	→ 12-week intervention, integrating approaches applied in Social Cognitive Theory (SCT): Intervention group: ◦Study website SHED-IT ◦Study handbook ◦Video content ◦Behavior change techniques targeting e.g., goal-setting, self-efficacy, depression, sleep, resistance training ◦Information of male specific research ◦Program messages ◦Mental fitness modules (text, interactive pictures, chats, quizzes) ◦Gradually introduced modules Control group: ◦Waitlist-control	Depression: •PHQ-9 •Beck Depression Inventory (BDI) •Male Depression Risk Scale (MDRS-22) Other: ◦Generalized Anxiety Disorder Questionnaire (GAD-7) ◦Weight, waist circumference, ◦BMI, body fat percentage ◦Blood measurements	Findings – Depression: •Group differences in favor of the intervention group for depression Findings – Other: Group differences in favor of the intervention group: ◦Weight Limited intervention effects on: ◦Cardio-metabolic effects Anxiety symptoms

### Risk of bias assessment

We used the Cochrane Collaboration’s Risk of Bias Assessment Tool (RoB 2) to assess the risk of bias of the included studies in this systematic review (50). The studies were rated as have ‘low risk’, ‘some concerns’, or ‘high risk’ of bias. The following quality domains were assessed: randomization process, deviations from intended interventions, missing outcome data, measurement of the outcome, and selection of the reported result (50).

## Results

### Main findings of the study

The outcomes of the study by Chang et al. (45) which included elements of the SCT in order to guide the intervention, showed that participants in the intervention group reported significant less depression symptoms than the control group. Results from two of the six included studies (48, 49) showed no significant group differences in depression symptoms. Jones et al. (46) who investigated whether participants’ mental health was associated with the rate of attendance and engagement with the behavioral weight management program (WW) and trial (WRAP) found that a reduction in depression symptoms was associated with higher participation rates in WW sessions. In addition, individuals with obesity who had fewer depression and anxiety symptoms at baseline were more likely to describe higher engagement with the program resources (WW e-tools and mobile phone app). Further, it can be concluded that higher attendance at all study visits up to 5 years follow-up were associated with fewer symptoms of depression. The outcomes of the study conducted by Young et al. (42), that focused on an online weight loss program targeted at men with low mood, showed significant improvements in depression symptoms. The findings of the study by Kim et al. (47) which investigated a new, comprehensive, multifactorial, daily, intensive psychologist coaching based on CBT modules, showed that digital CBT improved the level of depression symptoms. Baseline depression symptoms significantly predicted long-term clinical outcomes.

### Qualitative synthesis

#### Study selection

The systematic database search yielded 187 published studies (see **Figure 1**). After the screening process, 20 articles were included in the full-text assessment to check eligibility. Of the 20 full-texts, 14 publications were neither in line with the inclusion nor the exclusion criteria, resulting in six publications, which were ultimately included in the in-depth analyses and summary. All included RCTs had a two-arm design. A total of 2371 patients were included in these RCTs.

#### Summary of included studies

As presented in **Figure 1**, the search strategy initially yielded 214 records in PubMed, Scopus and Cochrane library. After removing duplicates and records deemed inappropriate, 112 articles remained to be screened. After screening these 112 articles, another 92 were excluded, leaving 20 articles. These articles were assessed for eligibility using the full text. Thus, after assessing the remaining 20 full-text articles, 14 additional articles were excluded due to a) study objectives that were primarily focusing on metabolic comorbidities, e.g., hypertension, hyperlipidemia, diabetes (*n* = 10), b) study objectives that were focusing primarily on eating disorders comorbidities e.g., binge eating disorder (*n* = 2) and c) study objectives that were focusing on pre- or post-bariatric surgery patients (*n* = 2). The final studies included in this review (*n* = 6) are listed in **Figure 1**.

#### Participants and study characteristics

The selected studies included patients from four different countries. Three studies included patients from the United States (42, 45, 48), one study included patients from Germany (49), one study included patients from England (46), and another study included patients from South Korea (47). Sample sizes ranged from 70 to 1267 participants (age range: 18-60 years). Participants included in the review had a mean BMI of 32.4 kg/m². Half of the studies included in the review (*n* = 3) focused on women. Two studies focused on men and one included both genders in their sample. The oldest included publication was conducted by Marcus et al. (48) in 2016 and tested the efficacy of a 6-month culturally adapted, individually tailored, Spanish-language Internet-based physical activity intervention among Latinas (*n* = 205). The second study was conducted by Chang et al. (45) and investigated whether a community-based intervention program that included video lessons and joined peer support group teleconferences had an effect on the self-efficacy to cope with stress, emotional coping response, social support for stress management, stress, depression, and positive and negative affect among mothers with obesity (*n* = 612). The third study was conducted by Kim et al. (47) and tested the efficacy of a comprehensive, multifactorial, daily intensive, coaching program by psychologists based CBT modules in weight management among female participants (*n* = 70). Three studies which were published in 2021 were included. The study conducted by Welzel et al. (49) implemented and evaluated an online tutorial with the aim of improving weight management and practitioner-patient interactions in primary health care settings among a sample of the German population (*n* = 185). The fifth included study was conducted by Young et al. (42) and explored the efficacy of a self-guided, eHealth program (SHED-IT: Recharge) to reduce weight and depression symptoms in men with obesity (*n* = 125). The final included study was conducted by Jones et al. (46) and examined whether mental health was associated with attendance and engagement in a trial of behavioral weight management programs among a sample of the British population (*n* = 1267).

#### Measurement of depression

The study conducted by Young et al. (42) was the only one of the six studies included in this review in which depression symptoms was the primary outcome of the study. The primary outcome of the majority of the studies included in this review were change in body weight and physical activity. Nevertheless, all studies made use of validated questionnaires to assess depression symptoms. To investigate depression symptoms in individuals with obesity, Young et al. (42) and Welzel et al. (49) made use of the Patient Health Questionnaire-9 (PHQ-9). The depression symptoms of participants in the studies of Chang et al. (45) and Marcus et al. (48) were measured using the Center for Epidemiologic Studies Depression Scale (CES-D). In addition, Chang et al. (45) integrated the Perceived Stress Scale (PSS) and the Positive and Negative Affect Scale (PANAS) to gain greater insight into the depression symptoms of individuals with obesity who participated in the intervention. The Hospital Anxiety and Depression Scale (HADS), also used in the study by Jones et al. (46) and the Beck Depression Inventory-II (BDI-II) were additional measurements used to determine depression symptoms in participants by Kim et al. (47).

#### EHealth approach

Welzel et al. (49) investigated the efficacy of a web-based 5A-based weight management counseling program for general practitioners in obesity treatment. The 5A program focuses on “ASK”, assessing health status, “ASSES”, counseling on the health benefits of treatment and available treatment options, “ADVISE”, seeking agreement on weight loss expectations, “AGREE”, agreeing on a treatment plan and goals, and “ASSIST”, assisting the patient in the process of weight management. Based on these 5As obesity management guidelines from the Canadian Obesity Network, a short 5A online tutorial was implemented. This online tutorial comprises an introduction in the beginning and five knowledge sections. Hereby, patients receive information from their primary care physicians in the form of online sheets about obesity-related treatment options (physical exercise, nutrition, psychotherapy, medication, and surgery). At the end of the 5A tutorial a short knowledge quiz was implemented.

The randomized controlled trial (RCT) by Chang et al. (45) was conducted to evaluate a community-based intervention program that integrated ten video lessons at home and participation in ten peer support group teleconferences. During the 16-week intervention, participants watched weekly video lessons (20 minutes per video lesson) about the topic stress management at home (weeks 1-4). From week five onwards until week 16, participants received video lessons on healthy eating and physical activity. After viewing the designated video lessons, participants circled responses on a worksheet that ask them about content of the video lessons. Moreover, they joined peer support group teleconferences (30 minutes per session, weekly for the first four weeks and then every other week for weeks 5-16). These group teleconferences were led by moderators who were peer educators or dietitians from the Special Supplemental Nutrition Program for Women, Infants, and Children (WIC), trained in motivational interviewing and group facilitation.

Marcus et al. (48) investigated the efficacy of a culturally and linguistically adapted, individually tailored, Internet-based physical activity intervention compared to a Spanish-speaking wellness contact Internet control group. Participants randomly assigned to the physical activity Internet intervention received access to a study website including the following features: 1) self-monitoring of minutes of activity and steps; 2) goal setting with graphs to compare goals to minutes recorded; 3) message board to foster social support among participants; 4) “Ask the Expert” where participants could anonymously ask questions to a PhD level researcher and 5) online resources such as walking route maps and free exercise videos. Next to these, participants were provided with monthly questionnaires that generated automated tailored physical activity reports. Participants assigned to the control group received access to a Spanish-language website with information on health topics other than physical activity. The web-based content focused on diet and other factors, that were associated with cardiovascular disease risk and integrated information from a series on heart health developed for Latinos by the National Heart Lung and Blood Institute.

Young et al. (42) explored whether a gender-tailored, self-guided, eHealth program (SHED-IT: Recharge Program) could help in the reduction of weight and depression in men with obesity with low mood. Hereby, participants took part in a 3-month program that included a combination of eHealth and print resources. At the very beginning, participants received a study handbook and watched two introductory videos on the study website. Participants were then encouraged to complete a series of tasks set out in the study logbook (e.g., setting and reviewing goals), which they received weekly. The focus of this program and the tasks included in it targeted behavior change techniques that align with key constructs of Bandura’s Social Cognitive Theory (SCT) (e.g., self-efficacy, goal setting) to achieve long-term lifestyle change in participants (51). In addition, the topics of sleep, resistance training, and four interactive “mental fitness” modules to combat depression were included. Young et al. (42) created these modules using the eHealth authoring program Articulate Rise. Each module was created with a set of tools such as text, interactive images and diagrams, quizzes on cognitive behavioral skills. Additionally, the program includes cognitive restructuring, mindfulness, behavioral activation, relapse prevention, and completion a thought monitoring diary.

Kim et al. (47) conducted an open-label, active-comparison RCT in order to investigate the efficacy of a psychologist-coaching program based on Cognitive Behavioral Therapy (CBT) modules via digital tools, such as the Noom Coach App and InBody Dial among 70 female participants. The Noom Coach App is a smartphone application, which allows to log food intake, exercise activity, weight, and engagement in in-app group activities, read in-app articles, and interact with a human coach via in-app messages. Moreover, a web-based dashboard is provided to the coaches to monitor participants’ data. The InBody Dial is a body composition measure that can be linked to a mobile app to conveniently measure the user’s body composition. The intervention group used the apps, linked to a therapist intervention via digital health care service that provided daily feedback and assignments for eight weeks. Whereas the control group also used the digital health care service, but practiced self-care without receiving the therapist’s intervention.

Jones et al. (46) randomly assigned 1267 individuals with obesity to either a brief intervention, Weight Watchers (WW) for 12-weeks, or WW for 52-weeks. Participants in the brief intervention received a 32-page printed booklet from the British Heart Foundation with self-help weight management strategies. Participation at WW included weekly WW sessions and engagement with digital tools, such as e-tools and online resources and use of the WW mobile phone app. The WW’s e-tools are an online service, integrating access to support materials (e.g., recipes, videos, community area) and tracking tools.

#### Risk of bias assessment

**Table 2** gives an overview of the risk of bias assessment of the included studies. In terms of the assessed domains, the randomization process was observed with some concerns in two studies, concerns regarding the deviations from intended interventions occurred in 2 studies. Regarding the other domains, no concerns/risk of bias could be overserved.

**Table 2:** Summary of the quality assessment.

	D1	D2	D3	D4	D5
Chang et al. (42)	?	?	+	+	+
Jones et al. (50)	+	+	+	+	+
Kim et al. (45)	?	+	+	+	+
Marcus et al. (43)	+	+	+	+	+
Welzel et al. (44)	+	?	+	+	+
Young et al. (39)	+	+	+	+	+

D1 = randomization process; D2 = deviations from intended interventions; D3 = missing outcome data; D4 = measurement of the outcome; D5 = selection of the reported result. Low risk = +, some concerns = ?, high risk = -.

## Discussion

The aim of this systematic review was to synthesize evidence from RCTs on the efficacy of eHealth interventions that target depression symptoms in individuals with obesity.

Due to emerging empirical evidence about depression symptoms experienced by individuals with obesity and the limited amount of eHealth interventions targeting this, a clear synthesis of this literature is of high relevance. Hence, a clear synthesis of the evidence relating to the efficacy of eHealth interventions targeting depression symptoms in individuals with obesity is of special relevance for the development of further interventions. So, the outcome of this review may assist in the development of further eHealth interventions that, specifically target the needs and demands of individuals with obesity and depression symptoms.

In total six RCTs were included in this systematic review. The overall quality of the studies included int his review was assessed as good. In general, the outcomes of the review showed that the majority of the included eHealth interventions were efficient in reducing depression symptoms in individuals with obesity. Nevertheless, the outcomes underlined the lack of interventions designed for individuals with obesity and depression symptoms as a primary outcome, based on the fact that only one the study of Young et al. (42) had depression symptoms as primary outcome. Hence, it is of high relevance for future studies and interventions to focus on this.

Besides that, it became apparent that study participants in the two interventions that did not show a significant difference in the depression symptoms experienced a limited user acceptance of the eHealth interventions (48, 49) compared to study participants in the other studies included in this review. This is in line with research which concluded, that the user acceptance for eHealth modalities is a vital precondition for such interventions to be effective and integrated in clinical settings (52). An additional factor that influences the efficacy of such eHealth interventions is the rate of attendance at the online sessions. Jones et al. (46) concluded that higher attendance rates correlated with lower levels of depression symptoms and higher life satisfaction scores. This underlines the outcomes of previous research emphasizing the association between attendance, engagement, and adherence in such interventions (53, 54).

Evaluation of the content of the different interventions showed that more variation in intervention content, modes of delivery and sequence of intervention messages correlated with lower post-intervention depressive scores (45). The outcomes of studies conducted by Chang et al. (45), Kim et al. (47), and Young et al. (42) showed that a multidimensional approach with human feedback and support based on elements of the CBT and SCT are most effective in the treatment of depression symptoms in individuals with obesity. Hereby, these interventions were fully tailored in multifactorial domains: the behavioral, cognitive, emotional, motivational, and physical domains. The majority of previous eHealth interventions focused on the mental health in individuals with obesity focused on diet, physical activity, weight loss or the combination of these (55–57). This emphasizes once again the complex reciprocal pathway between mental and bodily diseases and the importance of the treatment of both parts simultaneously in the treatment of individuals with obesity. On top of that, it underlines the relevance of the inclusion of cognitive features in obese treatment in order to achieve changes in the cognitive distortions, experienced by individuals with obesity. In contrast to such interventions, those focusing primarily on education through materials, i.e., non-interactive strategies showed less success in reducing depression symptoms (48, 49). Thus, interactive modules as used in the SHED-IT recharge intervention, conducted by Young et al. (42), that combined activities with educative materials, seemed to be particularly effective in the reduction of depression symptoms. The outcomes of this systematic review are in line with the outcomes of previously conducted meta-analyses and reviews, which showed that especially Internet-delivered Cognitive Behavioural Therapy (ICBT) is as effective as face-to-face therapy in the treatment of various mental disorders, such as depression, anxiety disorders and panic disorders (58, 59).

### Strengths and limitations of the present review

To the best of our knowledge, this is the first systematic review to summarize evidence on the efficacy of eHealth interventions specifically targeted at the treatment of individuals with obesity to reduce depression symptoms. This work was conducted in accordance with the PRISMA guideline for systematic reviews (44). Only RCTs were included, as this is the golden standard for efficacy research. We used broad search terms and searched several large databases, which still resulted in a small number of studies included in this review. The studies included in this systematic review are heterogeneous and disparate, which puts several limitations on the summarized findings. Generally speaking, only English studies were included in the study. On top of that, there are few eHealth interventions that focus on depression symptoms the mental health in individuals with obesity. The focus in this review was specified on individuals with obesity and depression symptoms, resulting in a limited amount of studies that could have been included in this review. This limits the generalizability of the present findings and obstructs the performance of a meta-analysis. In addition, approximately three-quarters of the samples in the reported studies consisted of women. Next to this, each study included in this review made use of a different depression assessment instrument. Thus, there is a lack of standardized measurement instruments among different studies. Therefore, the way depression symptoms was measured in the *N* = 2371 participants included in this review is not uniform which could lead to deviations.

## Conclusion

The evidence on interventions designed for individuals with obesity and depression symptoms as a primary outcome is very limited, only a small number of randomized controlled intervention studies have been published. The findings of this systematic literature review emphasize that eHealth interventions, targeting depression symptoms in individuals with obesity should be multidimensional. Furthermore, eHealth interventions that target both mental health, more specifically depression symptoms and physical health using interactive strategies are needed for better efficacy in meeting the needs of individuals with obesity. Especially eHealth interventions that included CBT modules or techniques and have integrated guided self-help approaches and digital tools seemed to have potentially high acceptance and efficacy in reducing depression symptoms. Hence, it can be concluded that future eHealth interventions that target depression symptoms in individuals with obesity should integrate CBT tools and techniques in combination with guided self-help approaches in order to enhance the efficacy of such interventions in reducing depression symptoms.

## Data availability statement

The original contributions presented in the study are included in the article/**Supplementary Material**. Further inquiries can be directed to the corresponding author.

## Author contributions

DK: Writing – original draft. AB: Writing – original draft. TS: Writing – original draft. JBK: Writing – original draft. SG: Writing – original draft. E-MS: Writing – original draft. MT: Writing – original draft.
