# Supplementary material for: Efficacy of eHealth interventions to reduce depression symptoms in individuals with obesity: a systematic review of randomized controlled trials
Source: Front Psychiatry. 2024 Mar 7;15:1296433. doi: 10.3389/fpsyt.2024.1296433 (PMC10954845; doi:10.3389/fpsyt.2024.1296433)
Supplement: Supplementary file 1 [file Table_1.docx]

| Database | Search strategy | Number of results |
| --- | --- | --- |
| Cochrane | (((obes*) AND (randomized controlled trial) OR (RCT) AND (depress*)) AND ((e-health) OR (mobile) OR (e-mental health) OR (web) OR (tele*)) AND (adult)) | N= 64 |
| PubMed | (((obes*) AND (randomized controlled trial) OR (RCT) AND (depress*)) AND ((e-health) OR (mobile) OR (e-mental health) OR (web) OR (tele*)) AND (adult)) | N= 94 |
| Scopus | (((obes*) AND (randomized controlled trial) OR (RCT) AND (depress*)) AND ((e-health) OR (mobile) OR (e-mental health) OR (web) OR (tele*)) AND (adult)) | N= 56 |
